# Paediatric pancreaticobiliary endoscopy: a 21-year experience from a tertiary hepatobiliary centre and systematic literature review

**DOI:** 10.1186/s12887-017-0959-9

**Published:** 2018-02-09

**Authors:** Margaret G. Keane, Mayur Kumar, Natascha Cieplik, Douglas Thorburn, Gavin J. Johnson, George J. Webster, Michael H. Chapman, Keith J. Lindley, Stephen P. Pereira

**Affiliations:** 1 Institute for Liver and Digestive Health, University College London, Royal Free Campus, Pond St, London, NW3 2PF UK; 2 Department of Gastroenterology, University College of London Hospital, 235 Euston Road, London, NW1 2BG UK; 3 Department of Gastroenterology, Great Ormond Street Hospital, London, WC1N 3JN UK

**Keywords:** Endoscopic retrograde Cholangiopancreaticography (ERCP), Endoscopic ultrasound (EUS), Paediatric, Chronic pancreatitis, Primary sclerosing cholangitis, Choledocholithiasis, Pancreatic fluid collection, Transmural drainage, Biliary leak, Cystic lesion of the pancreas

## Abstract

**Background:**

In adults ERCP and endoscopic ultrasound (EUS) are standard methods of evaluating and treating many hepatopancreaticobiliary (HPB) conditions. HPB disease is being diagnosed with increasing frequency in children but information about role of ERCP and EUS and their outcomes in this population remain limited. Therefore the aims of this study were to describe the paediatric ERCP and EUS experience from a large tertiary referral HPB centre, and to systematically compare outcomes with those of other published series.

**Methods:**

All patients <18 years undergoing an ERCP or EUS between January 1992–December 2014 were included. Indications for the procedure, rates of technical success, procedural adverse events and reinterventions were recorded in all cases.

**Results:**

Ninety children underwent 111 procedures (87 ERCPs and 24 EUS). 53% (48) were female with a median age of 14 years (range: 3 months - 17 years). Procedures were performed under general anaesthesia (*n* = 48) or conscious sedation (*n* = 63). Common indications for ERCP included chronic or recurrent pancreatitis and biliary obstruction. Patients frequently had multiple comorbidities, with a median ASA grade of 2 (range 1–4). Therapeutic procedures performed included biliary or pancreatic sphincterotomy, common bile duct or pancreatic duct stone removal, biliary or pancreatic stent insertion, EUS-guided fine needle aspiration and endoscopic transmural drainage of pancreatic fluid collections. No adverse events were reported following ERCP but there was one complication requiring surgery following EUS guided cystenterostomy.

**Conclusion:**

ERCP and EUS in children and adolescents have high technical success rates and low rates of adverse events when performed in high volume HPB centres.

## Background

In adult populations endoscopic retrograde cholangiopancreatography (ERCP) and endoscopic ultrasound (EUS) are commonly used in the diagnosis and management of many hepatopancreaticobiliary (HPB) conditions [1, 2]. Pancreaticobiliary disorders are being diagnosed with increasing frequency in children [3, 4]. This is probably as a result of a rise in predisposing risk factors for HPB disease as well as improvements in the sensitivity and availability of diagnostic tools to detect these conditions. However, EUS and ERCP in this population continue to be performed relatively rarely [Table 1], which may be due to a lack of awareness of the indications or limited local availability of advanced endoscopists who are able to perform these procedures in this population [5].

**Table 1:** Indications and outcomes from paediatric ERCP and EUS case series published between 2000 and 2015

Author and year	Number of patients	Adult or paediatric endoscopist	Number of procedures	Procedure type	Indication for ERCP / EUS	Technical success % (n)	Therapy performed % (n)	Adverse events % (n)
Hsu RK 2000 [7]	22	Adult	34	ERCP	AP (6), recurrent pancreatitis (5), CP (11)	NR	53% (18)	6% (2)
Poddar U 2001 [8]	72	Paediatric	84	ERCP	Suspected biliary tract disease (44), suspected choledochal cysts (14), extrahepatic portal venous obstruction before shunt surgery (19), suspected CBD stone (2), histiocytosis with cholestatic jaundice (2), bile leak (2), autoimmune hepatitis (1), CP or recurrent pancreatitis (14), pancreatic ascites or fistula (6), recurrent abdominal pain (8)	97% (70/72)	30% (22)	8% (6)
Prasil P 2001 [9]	20	Adult	21	ERCP	Biliary indication (15), pancreatic indication (6)	91% (19)	48% (10)	33% (7)
Varadarajulu S 2004 [29]	116	Adult	163	ERCP	Suspected biliary obstruction (47), Bile leak (9), Pancreatitis (acute gallstone, CP, recurrent) (58), traumatic PD injury (2)	98% (161)	67% (77)	2.5% (3)
Cheng CL 2005 [10]	245	Adult	329	ERCP	Biliary pathology (93), pancreatic pathology (111), abdominal pain, suspected biliary or pancreatic origin (41)	98% (322)	71% (235)	9.7% (32)
Varadarajulu S 2005 [36]	14	Adult	15	EUS	AP or recurrent pancreatitis (6), suspected biliary obstruction (5), abdominal pain (3)	100% (15)	0% (0)	0% (0)
Durakbasa CU 2008 [11]	28	Adult	32	ERCP	Biliary pathology (21), pancreatic pathology (7)	100% (32)	63% (20)	6% (2)
Cohen S 2008 [37]	32	Adult	32	EUS	Recurrent pancreatitis (9), pancreatic mass (6), obstructive jaundice (4), oesophageal stenosis (4), oesophageal mass (2), duodenal indication (1), stomach and rectal indication (2)	100% (32)	0% (0)	0% (0)
Attila T 2009 [38]	38	Adult	40	EUS	Pancreatitis (10), solid pancreatic mass (7), cystic pancreatic mass (1), CP + cyst (1), suspected annular pancreas (1), coeliac plexus block (1), CBD stone (1), abdominal pain and atrophic pancreas (1), ampullary adenoma (1), abnormal MRCP (1)	100% (40)	5% (2) Coeliac plexus block	0% (0)
Vegting IL 2009 [12]	61	Adult	99	ERCP	Biliary atresia (26), choledochal cyst (7), cholestasis (6), CBD stone (5), Bile leak post surgery (4), traumatic liver injury (1), PSC (1), portal cavenoma (1), pancreatitis (4), traumatic pancreatic injury (3), pseudocyst (2), pancreaticoblastoma (1)	100% (99)	60% (60)	4% (4)
Li ZS 2010 [39]	42	Adult	110	ERCP	CP (42)	100% (110)	100% (110)	17.3% (19)
Jang JY 2010 [13]	122	Paediatric^a^	245	ERCP	AP (7), recurrent AP (11), CP (20), trauma (3), pancreatic mass (2), choledochal cysts (40), choledocholithiasis (24), suspected sclerosing cholangitis (8), trauma (2), other (4)	98% (241)	78% (190)	18% (45)
Otto AK 2011 [14]	167	Adult	231	ERCP	CP or recurrent pancreatitis (106), AP (42), CBD stone (26), choledochal cyst (2), congenital biliary obstruction (2), malignant biliary obstruction (1)	100% (231)	69% (159)	4.7% (11)
Troendle DM 2013 [40]	65	Paediatric	65	ERCP	Choledocholithiasis (65)	98% (64)	100% (100)	8% (5)
Enestvedt BK 2013 [41]	296	Adult	429	ERCP	Abnormal liver-associated enzymes (109), CBD stone (107), follow-up ERCP (52), recurrent pancreatitis (47), suspected bile or pancreatic duct leak (45), cholangitis (27), jaundice (23), abdominal pain (12), cyst drainage (4), PD endotherapy (3)	95% (408)	64% (275)	7.7% (33)
Limketkai BN 2013 [24]	154	Adult	289	ERCP	CBD stone (52), elevated transaminases (25), PSC (16), stent removal (12), cholangitis (7), CBD stricture (7), bile leak (6), choledochal cyst (7), chronic abdominal pain (8), recurrent pancreatitis or CP (110), stent removal (33), pseudocyst (18), PD stone (14), AP (9), PD stricture (3), PD disruption (2), pancreatic mass (2), post-operative pancreatic fistula (2)	94% (272)	86% (247)	5.9% (17)
Halvorson L 2013 [42]	45	Adult	70	ERCP	CBD stone (17), PD leak (5), bile duct leak (12), PD stricture (1), CBD stricture (3), pancreas divisum (5), CP (1), pseudocyst (2), ampullary adenoma (1), SOD (2), recurrent AP (5), PSC (2)	99% (69)	100% (70)	7.1% (5)
Agarwal J 2014 [1]	172	Adult	221	ERCP	Abdominal pain (153), pancreatic fistula (11), symptomatic pseudocyst (4), and jaundice (3)	100% (221)	71% (157)	4.7% (8)
Kieling CO 2014 [43]	60	Adult	75	ERCP	Bile duct obstruction (49.3%), sclerosing cholangitis (18.7%), post-surgery complication (12%), biliary stent (10.7%), choledochal cyst (5.3%), and pancreatitis (4%)	95% (57)	73% (55)	9.7% (7)
Liu W 2014 [44] (Abstract)^b^	68	Unknown	102	ERCP	Bile duct stone (37), PD stone (8), bile duct benign stricture (7), other (16)	100% (102)	NR	4% (4)
Oracz G 2014 [45]	157	Adult	481	ERCP	CP (481)	99% (475)	46% (223-PD stent)	1.9% (9)
Saito T 2014 [46]	220	Paediatric	235	ERCP	Choledochal cyst (92), biliary atresia (62), other (cholelithiasis, hepatitis, pancreatitis, choledocholithiasis) (66)	96% (225)	3% (8)	9.8% (23)
Scheers I 2015 [5]	48	Adult	52	EUS (+/− combined ERCP)	Suspected biliary obstruction (20), AP or CP (20), pancreatic mass (3), pancreatic trauma (7), ampullary adenoma (2)	98% (51)	25% (13)	3.8% (2)
Giefer MJ 2015 [47]	276	Adult	425	ERCP	Biliary obstruction (184) CP (114), suspected SOD (38), AP (29), relapsing pancreatitis (8), other (52)	95% (403)	81% (346)	8.8% (28)
Ford K 2015 [48]	9	Adult	9	ERCP	CP (9)	78% (7)	78% (7)	0% (0)

*ERCP* endoscopic retrograde cholangiopancreaticography, *EUS* endoscopic ultrasound, *NR* not recorded, *CBD* common bile duct, *MRCP* magnetic resonance cholangiopancreatography, *PD* pancreatic duct, *SOD* sphincter oddi dysfunction, *AP* acute pancreatitis, *CP* chronic pancreatitis, *PSC* primary sclerosing Cholangitits

^a^= adult endoscopist supervision in initial and complex cases

^b^=article in Chinese, English abstract only

ERCP may be associated with adverse events, such as acute pancreatitis in approximately 3.5% of unselected adult patients [6]. The frequency of these events depends on the indication for the procedure, the patient and their comorbidities and the experience of the endoscopist. In a paediatric population some case series have reported much higher rates of adverse events of up to 33% [Table 1] [7–16]. With greater availability of alternative diagnostic investigations such as magnetic resonance imaging or EUS, almost all ERCPs in adults are now performed for therapeutic indications as advocated by the American Society for Gastrointestinal Endoscopy [17]. Such guidelines do not exist for the paediatric population, but a recent study from the USA has shown that although the annual numbers of paediatric ERCPs being performed is rising, of late they are almost always being undertaken for therapeutic indications [4].

Over the last 30 years in adults, the indications for diagnostic and therapeutic EUS have expanded significantly. For solid and cystic lesions of the pancreas, EUS is recognised to be a sensitive method of diagnosing features of malignancy as well as enabling simultaneous tissue sampling for cytological or histopathological analysis [18]. In biliary obstruction, EUS is the most sensitive test for diagnosing choledocholithiasis and can also enable evaluation and sampling of biliary strictures [19, 20]. EUS is also used in a growing number of therapeutic applications such as the drainage of symptomatic pancreatic fluid collections (PFC) [19, 21, 22]. Experience of EUS in a paediatric population along with outcomes and long-term follow-up is particularly limited [Table 1].

## Methods

### Study aim

The primary aim of this study was to determine the indications and outcomes for ERCP and EUS in a paediatric population referred to a high-volume tertiary referral HPB centre. A secondary aim was to conduct a systematic review of the literature from January 2000 to June 2015 and compare indications, rates of technical success and adverse events to other published case series.

### Design

Retrospective cohort study and systematic literature review.

### Setting

A large regional HPB centre. Endoscopic procedures were performed at Great Ormond Street Hospital (GOSH) or University College London Hospital (UCLH). Surgical procedures were performed at UCLH or the Royal Free Hospital, London or GOSH.

### Inclusion criteria

Patients <18 who underwent an ERCP or EUS between January 1st 1992 and December 31st 2013.

#### Data recorded

Cases were identified primarily from records of the HPB multidisciplinary team meetings, which are held weekly. In addition, the Pathology (CoPath histology database, Sunquest, Tucson AZ, USA), Endoscopy (GI reporting tool, Unisoft medical systems, UK) and Imaging (PACS: picture archiving and communication system, GE Healthcare, USA) databases were searched.

The electronic medical records of the included patients were reviewed and information was recorded in an electronic spreadsheet. Data collected included demographic information (age, sex, hospital number), initial symptoms, and history of acute or chronic pancreatitis or malignancy, family history of pancreatic cancer or relevant clinical syndrome. Cross-sectional imaging (computed tomography (CT) and/or magnetic resonance cholangiopancreatography (MRCP)) features were recorded. Details of the endoscopic procedure along with cytology, histology or culture results where available were also recorded. For patients ultimately referred for surgery, date of the operation, type of resection and final histology were recorded. Length of follow-up was calculated from first procedure to last clinic appointment attended, or date of clinic discharge, or death.

### Definitions of outcomes and adverse events

Technical success at ERCP was defined as successful deep cannulation of the desired duct and completion of the therapeutic aim.

Technical success at EUS was defined by successful visualisation of the desired area of the gastrointestinal tract and in therapeutic EUS by completion of the therapeutic aim.

Adverse events following ERCP or EUS were defined as sphincterotomy bleeding (in which blood transfusion or endoscopic therapy was required for management); perforation; post-ERCP pancreatitis, defined as abdominal pain associated with a serum amylase 3 times the upper limit of normal (stratified by the Cotton severity criteria: mild, 0–3 days of hospital stay; moderate, 4–10 days of hospital stay; severe, >10 days of hospital stay); cholangitis (fever and biliary symptoms in the absence of concomitant infection; other source of sepsis which prolonged inpatient stay and death.

### Procedures

#### Endoscopic retrograde cholangiopancreatography (ERCP)

Informed written consent for the procedure was obtained from the parent or guardian of each child. The procedures were performed under general anaesthesia or conscious sedation with midazolam and fentanyl. All ERCPs were performed by one of four experienced endoscopists using a standard adult diagnostic or therapeutic duodenoscope (JF; Olympus, Southend-on-Sea, UK). All procedures were performed in the endoscopy unit with fluoroscopy. Sphincterotomy was performed using standard accessories (Cook Medical or Boston Scientific). Stones were extracted with standard baskets or extraction balloons (Olympus or Cook Medical). All patients were observed for 4 h in the recovery area prior to discharge. Those with significant comorbidity or who became symptomatic following the procedure were admitted to hospital for further observation and management as needed.

#### Endoscopic ultrasound (EUS)

Informed written consent for the procedure was obtained from the parent or guardian of each child. The procedures were performed under conscious sedation or general anaesthesia using a radial or linear array echoendoscope (Olympus, UK). In children under 1 year of age, an endobronchial ultrasound (EBUS) scope was used. Fine-needle aspiration (FNA) was performed using either a 19 or 22 gauge FNA needle (Cook Medical or Boston Scientific) and biopsies were performed using a 19 or 22 gauge ProCore needle (Cook Medical).

For endoscopic transmural drainage of pancreatic fluid collections (PFC), EUS guidance was used to ensure the distance between the gastric and/or duodenal wall and the PFC was <1 cm and there were no interposed blood vessels on Doppler. PFC were generally accessed from the stomach using a cystotome (Cook Medical), alternatively a 19 gauge access needle (ECHO-19; Cook Medical) was used. Entry was confirmed by aspiration of cyst contents, after which two 0.035 in. guidewires were then advanced into the PFC and allowed to coil within the cyst under fluoroscopic guidance, which was used in all cases. The tract was then dilated with a controlled radial expansion (CRE) or Hurricane RX wire-guided balloon (Boston Scientific) or Soehendra biliary dilator. Usually two double-pigtail stents (7F–10F) of various lengths were then inserted into the fistulotomy using a Teflon pusher catheter (Cook Medical). Cyst fluid was obtained and sent for Gram stain, culture, and fluid amylase levels as clinically indicated. Patients were discharged when clinically stable (aim within 24 h) and prescribed a short course of oral antibiotics for up to 5 days. They were then followed up in clinic 3–6 monthly as necessary. Transmural stents were removed at 9–12 months if the PFC had resolved on cross-sectional imaging. If patients remained symptomatic, and the PFC persisted or recurred, additional drainage was performed following discussion at the HPB multidisciplinary team (MDT) meeting.

### Data analysis

Statistical Package for Social Sciences for Windows, version 18.0 (SPSS Inc., Chicago, IL, USA) was used to perform all statistical analyses. Associations between various clinical and radiographic characteristics were evaluated using a 2-sample *t* test for continuous variables, and a Chi-squared test for categorical variables.

### Systematic review

**Fig. 1:**
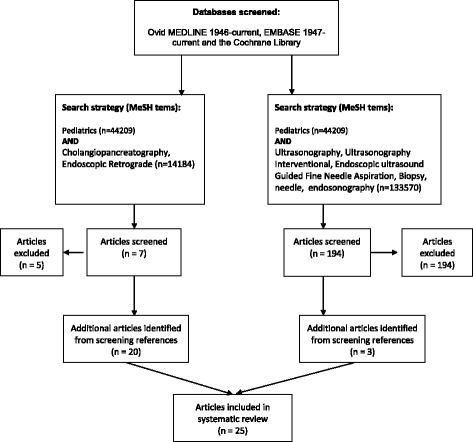
Systematic literature review flowchart

A systematic literature search was performed using the PubMed, EMBASE databases and the Cochrane Library for studies published in the English language between 1960 and 2015 and was conducted in accordance with the PRISMA guidelines [23]. MeSH terms were decided by a consensus of the authors and were restricted to the title, abstract and keywords. Articles that described outcomes in fewer than 5 patients were excluded. Case reports, abstracts, and reviews were also excluded. All references were screened for potentially relevant studies not identified in the initial literature search. The following variables were extracted for each report when available: Author and year, number of patients, number of procedures, procedure type, performed by a paediatric or adult gastroenterologist, indication for ERCP / EUS, technical success, proportion in which therapy was performed and adverse events. Twenty-five papers were included in the final analysis [Fig. 1, Table 1].

## Results

### Systematic review of the literature

**Fig. 2:**
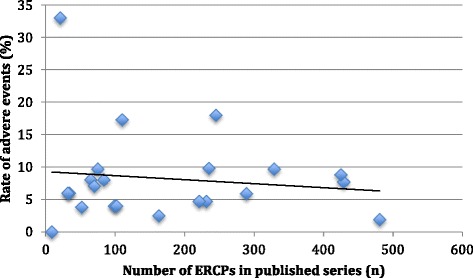
Reported adverse events compared to number of cases reported in each published series in the systematic review for paediatric ERCP

The results of the systematic review of the literature are outlined in Table 1. The lowest rates of adverse events were seen in ERCP procedures performed for chronic pancreatitis, EUS procedures compared to ERCP procedures (mean rate of adverse event: 0.95% vs. 8.4% respectively), or if the procedure was performed by an adult endoscopist compared to a paediatric endoscopist (mean rate of adverse events 6.64% vs. 10.95% respectively). A trend to lower rates of adverse events was also seen in published series, which reported higher number of cases [Fig. 2].

### Univeristy College London experience

**Fig. 3:**
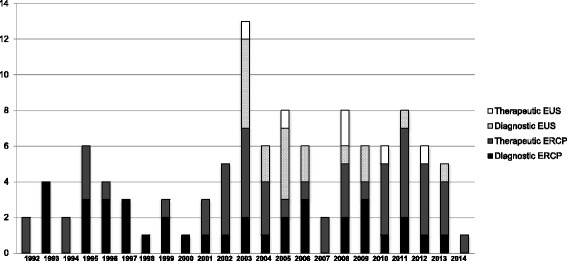
Number of ERCP and EUS procedures performed per year during the study period

During the 21-year study period the number of pancreaticobiliary procedures performed increased annually. The proportion of diagnostic ERCP procedures decreased, but numbers of therapeutic ERCP and EUS procedures increased [Fig. 3].

### ERCP

#### Patient demographics

During the 22 year study period, 66 patients had 87 ERCPs (median age of 14 (range 3–17) years). 52% (34) of patients were female. Procedures were undertaken for chronic or recurrent pancreatitis (48), biliary obstruction (34 patients – 29 with suspected choledolithiasis and 5 with a biliary stricture), cystic lesion of the pancreas (3) and suspected postoperative biliary leak (2). 62% of procedures were performed under general anaesthesia. Patients had a median ASA grade of 2 (range 1–4) due to a range of comorbidities [Table 2].

**Table 2:** Comorbidities of patients by indication for advanced endoscopy

Procedure and indication	Predisposing factors for pancreaticobiliary disease	Additional comorbidities
ERCP		
Biliary obstruction (stricture and stone disease)	Thalassaemia intermedia with chronic haemolysis, short gut syndrome, cholecystitis, hyperbilirubinaemia, hereditary spherocytosis, spine abscess requiring fusion of vertebrae and prolonged course of ceftriaxone, meningococcal septicaemia requiring ceftriaxone, sickle cell disease, anaplastic large cell lymphoma, multiple endocrine neoplasia type 1 with insulinoma requiring pancreatic resection	Eosinophilic gastroenteropathy, premature birth, patent ductus arteriosis repair, Salmonella septicaemia, learning difficulties, pyruvate kinase deficiency, pneumonitis
Bile leak		
Chronic or recurrent pancreatitis	Juvenile onset chronic pancreatitis, hereditary pancreatitis, cystic fibrosis, gallbladder stones, Caroli’s disease, chronic pancreatitis, Senior loken syndrome, chronic granulomatous disease, bone marrow transplant	Intestinal atresia, autoimmune enterocolitis, polycystic kidney disease, duplex kidney, renal transplant, left ventricular outflow tract obstruction, laparoscopic cholecystectomy, type 1 diabetes, bilateral carpal tunnel, autonomic dysfunction, postural hypotension, Lebers amaurosis, joint surgery, functional neurological and bowel disorder, deep vein thrombosis,
Pancreatic fluid collection		
EUS		
Diagnostic	Acute pancreatitis, chronic pancreatitis, lymphoma (Hodgkin’s, anaplastic large cell or T- cell non Hodgkin’s), hereditary spherocytosis, Beckwith Wiedman Syndrome,	Alpha 1-antitrypsin deficiency, liver transplant, epilepsy, pneumonitis, avascular necrosis of the hip, vascular occlusive disease, hiatus hernia, irritable bowel syndrome, Rhabdomyoma
Therapeutic	Hereditary pancreatitis, chronic pancreatitis, acute pancreatitis, Lebers amaurosis, Caroli’s disease, Senior loken syndrome,	Functional neurological and bowel disorder, duplex kidney, deep vein thrombosis, renal failure, laparoscopic cholecystectomy, joint surgery

#### Therapeutic interventions by indication

##### Chronic or recurrent pancreatitis

Thirty-two patients (median age 13 (range 5–17) years) had 48 ERCPs for the management of chronic or recurrent pancreatitis. The procedure was feasible in all but one case, with a pancreatogram being successfully obtained in 47 cases and a cholangiogram in 39. ERCP findings were as follows; chronic calcific pancreatitis in 42 cases, partial or complete pancreatic divisum in 4 cases (3 with concomitant calcific chronic pancreatitis) and choledochal cysts in 5.

**Fig. 4:**
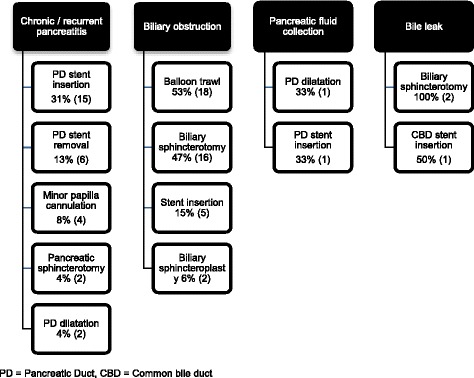
Therapeutic interventions performed for each diagnostic indication

Access was obtained via the major papilla in all cases and a biliary sphincterotomy was performed in 4 cases (one combined with a sphincteroplasty). A pancreatic sphincterotomy was performed in 3 cases, all undertaken from the major ampulla, using a standard wire guided sphincterotome in two cases and a minitome double lumen sphincterotome in the other case (Cook Medical, Limerick, Ireland). Cannulation of the minor papilla was attempted in 4 cases and was successful in 3 using a Cramer metal tipped catheter (Cook Medical, Limerick, Ireland). A single pigtail or straight therapeutic pancreatic stent was removed in 6 cases and inserted in 15 cases (5-7Fr and 4-7 cm in length). Therapeutic pancreatic stents were typically left in place for 4–6 months. Two patients had a pancreatic duct stricture dilated with Soehendra dilators [Fig. 4]. The procedure was combined with endoscopic transmural drainage of a PFC in one patient and percutaneous endoscopic gastrostomy (PEG) tube insertion in another. 63% (30) of patients were discharged on the same day as the procedure. No major complications occurred following the procedure but four of the 11 patients receiving conscious sedation required reversal agents (flumazenil or naloxone). Mean follow-up was 50 (range 0–232) months; 21 patients required a further EUS or ERCP and two patients underwent surgery for chronic pancreatitis (one had a Berger procedure and the other a distal pancreatectomy).

##### Biliary obstruction

Thirty-four ERCPs were undertaken for biliary obstruction; 29 for suspected choledocholithiasis and 5 for a biliary stricture. An ERCP was feasible in all but one case when the patient could not tolerate the procedure under conscious sedation and it was rescheduled under general anaesthesia. In patients with suspected choledocholithiasis a cholangiogram was performed in all cases and a pancreatogram in 8. A biliary sphincterotomy was performed in 16 cases and a sphincteroplasty in 2 cases (one in combination with a small sphincterotomy). A balloon trawl was performed in 18 cases and stones or sludge were removed in 11. In 3 cases complete stone clearance was not achieved and a biliary stent was placed [Fig. 4]. Mean follow up was 17 months (range 0–177); 8 patients required a further ERCP, EUS or percutaneous transhepatic drainage. All patients were referred for consideration of a laparoscopic cholecystectomy following bile duct stone clearance.

In the 5 patients with a biliary stricture a cholangiogram was performed in all cases and a pancreatogram in 2 cases. The strictures were due to lymphoma (1), pancreatitis (1) and of unknown aetiology (3). A biliary sphincterotomy or sphincteroplasty was not required in any case. Stricture dilatation was attempted with Soehendra dilators in one case and in two patients a biliary stent was inserted. 59% (20) of patients were discharged on the same day as the procedure. Mean follow up was 12 (range 0–57) months; 2 patients required subsequent percutaneous transhepatic drainage or EUS to further evaluate the stricture. No procedure-related complications were observed but one patient died within 9 days of the ERCP due to progression of lymphoma.

##### Pancreatic fluid collections

Three patients had an ERCP for a pancreatic fluid collection (indeterminate cystic lesion (1), pseudocyst (1) and suspected pancreatic duct leak (1)). A pancreatogram was obtained in all cases and a cholangiogram in one case. In the patient with an indeterminate pancreatic cyst a diagnostic EUS was also performed immediately after the ERCP. The inflammatory PFC had concomitant EUS-guided transmural drainage in addition to dilation of a pancreatic duct stricture and insertion of a pancreatic stent at ERCP. Following the procedure the cystgastrostomy stents migrated leading to a pneumoperitoneum, which required an exploratory laparotomy (further details are outlined in the therapeutic EUS section below).

##### Bile leak

Two patients had an ERCP for a post-operative bile leak; one following a laparoscopic cholecystectomy that resolved after a standard biliary sphicterotomy and biliary stent insertion [Fig. 4]. In a patient with a bile leak following hepatic resection, biliary access could not be achieved despite a needle knife sphincterotomy being performed at the time of ERCP, but the leak later resolved spontaneously. No complications occurred following either ERCP.

### EUS

#### Patient demographics

Twenty-four patients with a median age of 14 years (range: 3 months to 17 years) underwent an EUS. 67% (16) were female. Eighteen of the procedures were diagnostic and 6 therapeutic. Nineteen procedures were performed under general anaesthesia and 5 with conscious sedation.

#### Diagnostic EUS

The most common indication for EUS was to evaluate the pancreas (*n* = 9). Other indications included: evaluation of biliary obstruction (*n* = 4), mediastinal EUS (*n* = 3), evaluation of a gastric polyp (*n* = 1), and exclusion of oesophageal varices prior to laparoscopic fundoplication (*n* = 1).

Ten procedures were performed with a radial echoendoscope, five with a linear echoendoscope, two with both a radial and a linear echoendoscope and one with an EBUS scope in a 3-month-old child. Fine needle aspiration (22G needle) was performed in 6 cases with a biopsy (19G Procore needle) in 2 cases. Four samples were taken from mediastinal lymph nodes and 2 from solid pancreatic masses. Cytology was diagnostic in 67% (4) of cases. Histology was non-diagnostic in both cases. 56% (10) of patients having a diagnostic EUS were discharged the same day. Mean follow up was 49 months (range 0–1332); one patient died nine days after their procedure due to disease progression (recurrent lymphoma) and 2 patients subsequently required surgery (laparoscopic cholecystectomy and distal pancreatectomy and splenectomy).

#### Therapeutic EUS

Of the six patients that underwent therapeutic EUS, five had EUS guided transmural drainage (ED) of a PFC and one patient had a coeliac plexus block. The coeliac plexus block was performed in a 17 year old female who had hereditary chronic pancreatitis with abdominal pain requiring long-term transdermal and oral opioids. The procedure was performed under general anaesthesia with the patient in the supine position. The coeliac axis was located with a linear EUS scope and 10 ml of 0.25% bupivacaine and 100 mg triamcinolone were injected adjacent to the coeliac artery with no immediate complications. The patient reported little improvement in her pain symptoms following the procedure and continued on her previous medications.

The five patients undergoing ED of a symptomatic PFC had a median age of 14 years (range 9–17 years). Two patients had a PFC secondary to acute pancreatitis (cause unknown), the other three were due to chronic pancreatitis (one case was thought to be secondary to intrahepatic stones causing recurrent and ultimately chronic pancreatitis, the cause was unknown in the other two cases). The procedure was performed under GA in 4 of the 5 cases. ED was technically successful in all cases. Two patients had concomitant ERCP and pancreatic stent insertion. All patients having EUS-guided therapy were admitted for observation following the procedure. Mean follow-up was 30 months (range 1–59); one patient required flumazenil (the only EUS cyst enterostomy performed under conscious sedation) and one patient developed a pneumoperitoneum due to stent migration and required an exploratory laparotomy. At laparotomy the stents were noted to have migrated into the lesser sac, which contained pus and was lavaged. The cyst was then deroofed and a cystjejunostomy created following repair of the posterior stomach wall. After some delays in wound healing, the patient was discharged from hospital in good health 2 weeks after admission. No further problems were reported when the patient was last seen in outpatients, 14 months after the procedure.

## Discussion

Therapeutic ERCP and EUS can have a significant impact on the management of children with a range of HPB conditions, offering a minimally invasive, often day-case alternative to surgical treatment in many situations. However previous case series have reported adverse events in up to 33% after ERCP, [7–16] with pancreatitis being the most common event [9, 12, 15]. In this study no adverse events were observed following ERCP. In some series rates of post-ERCP pancreatitis and adverse events have been reported to be higher in those less than 6 years of age [24] and when procedures are performed in low volume or non-HPB centres [25, 26]. The good outcomes observed in this study may reflect that the population contained a high proportion of patients with chronic pancreatitis, very few children under the age of 6 and that all procedures were performed by experienced adult endoscopists working in a HPB centre, who routinely perform more than 150 ERCPs/year. Comparable results have been reported from other high volume adult and paediatric HPB centres [Table 1], thus supporting emerging recommendations for all paediatric pancreaticobiliary endoscopy to be carried out in high volume units.

For adult patients with painful chronic pancreatitis and a dilated pancreatic duct, endoscopic PD decompression by stricture dilatation, removal of PD stones and/or and pancreatic stent placement can improve symptoms although surgical drainage is associated with better long-term outcomes [27]. In a recent series of 143 paediatric patients who underwent therapeutic pancreatic ERCP for chronic pancreatitis, rates of analgesic use dropped significantly following the procedure [1]. In this series only 6% of patients undergoing ERCP for chronic pancreatitis ultimately required pancreatic surgery.

Pancreatic pathology in children differs to adults with a much lower incidence of premalignant and malignant lesions. In this study very few patients had an EUS for a primary pancreatic indication and more than a third of patients had tissue sampling or EUS-guided therapy at the time of the procedure. EUS-FNA (*n* = 6) and coeliac plexus block (*n* = 1) were safe and effective with no associated complications. The diagnostic yield for cytology following FNA was 67%, which is comparable to reports from adult populations [28].

In adults, EUS-guided drainage of a PFC is increasingly the preferred method for draining PFCs. In comparison to surgical drainage it has been associated with comparable rates of technical success, lower rates of adverse events and shorter hospital stays [29, 30]. In this study 5 paediatric patients underwent EUS-guided ED. The procedure was technically successful in all cases although one patient did develop a pneumoperitoneum requiring laparotomy. Rates of technical success and adverse events following ED were comparable to that seen in an adult series, which included a proportion of complex cases (e.g. walled off pancreatic necrosis or portal hypertension) [30–33].

### Study limitations

The main limitations of this study are that the data were collected retrospectively and the procedures were undertaken in a high-volume adult tertiary referral HPB centre (performing more than 3000 ERCP / EUS procedures annually with low rates of adverse events in the adult population: <5% in high risk therapeutic ERCPs [34] and <10% in therapeutic EUS [30]); therefore outcomes may not be generalisable to all endoscopy units. Although this series is smaller than some International series from adult and paediatric centres [Table 1], it does represent the largest UK experience to date. In many cases patients were transferred back to their original hospital after recovery from their endoscopic procedure, so medium to late onset complications (e.g. pancreatitis) may have been underestimated. Authors of other series performed in adult centres have reported similar limitations [1].

## Conclusions

In summary, ERCP and EUS in children and adolescents undertaken for similar indications, had comparable outcomes to adults and were associated with low rates of adverse events when the procedure were performed in high-volume HPB centres.

## Abbreviations

ASA: American Society of Anesthesiologists
CBD: Common bile duct
CRE: Controlled radial expansion
CT: Computed tomography
EBUS: Endobronchial ultrasound
ED: EUS guided transmural drainage
ERCP: Endoscopic retrograde cholangiopancreaticography
EUS: Endoscopic ultrasound
FNA: Fine-needle aspiration
GOSH: Great Ormond Street Hospital
HPB: Hepatopancreaticobiliary
MDT: Multidisciplinary team
MRCP: Magnetic resonance cholangiopancreatography
NIHR: National Institute for Health Research
NR: Not recorded
PACS: Picture archiving and communication system
PD: Pancreatic duct
PEG: Percutaneous endoscopic gastrostomy
PFC: Pancreatic fluid collections
PSC: Primary sclerosing cholangitits
SOD: Sphincter oddi dysfunction
UCLH: University College London Hospital
